# Pre-Growth Environmental Stresses Affect Foodborne Pathogens Response to Subsequent Chemical Treatments

**DOI:** 10.3390/microorganisms10040786

**Published:** 2022-04-08

**Authors:** Amandeep Singh, Veerachandra Yemmireddy

**Affiliations:** 1 School of Earth, Environmental and Marine Sciences, University of Texas Rio Grande Valley, 1201 W University Dr, Edinburg, TX 78539, USA; amandeep.singh01@utrgv.edu; 2 Department of Biology, University of Texas Rio Grande Valley, 1201 W University Dr, Edinburg, TX 78539, USA

**Keywords:** *Salmonella*, *Listeria monocytogenes*, *E. coli* O157:H7, environmental stress, pre-growth condition, sanitizer, sub-lethal injury

## Abstract

Foodborne pathogens such as *Salmonella*, *E. coli* O157:H7, and *Listeria monocytogenes* are known to survive under different environmental stresses with an effect on their physiological properties. The purpose of this study was to determine the effect of different environmental stresses on the foodborne pathogens response to subsequent chemical treatments. Three types of pathogens *Salmonella*, *E. coli* O157:H7, and *Listeria monocytogenes* were subjected to different environmental stresses: (i) Desiccation (ii) high salt (iii) low pH, and (iv) temperatures (14, 23, and 37 °C) during their growth. The cells harvested at their early stationary growth phase were subsequently subjected to chlorine (100 or 200 ppm), peracetic acid (40 or 80 ppm), and 0.5% lactic acid treatments. The results showed that pre-growth stress conditions have significant effect on the reduction of tested pathogens depending upon the type of chemical treatment. *Salmonella* showed the highest sensitivity against all these treatments when compared to *E. coli* O157:H7 and *Listeria monocytogenes*. In addition, *Listeria monocytogenes* showed the highest percentage of sub-lethally injured cells. These findings highlighted the need to consider pre-growth conditions as an important factor for the validation of physical and chemical intervention treatments.

## 1. Introduction

Foodborne bacterial pathogens such as *Salmonella*, *Escherichia coli* O157:H7, and *Listeria monocytogenes* are frequently implicated in several multi-state outbreaks in different food commodities and products. For example, these foodborne illnesses cost approximately $15.6 billion a year and one-third of total outbreaks are associated with fresh produce [1]. Several pre- and post-harvest factors contribute to the survival, growth and/or death of organisms in the food processing environment including but not limited to fresh produce operations. One of the reasons that these organisms are well connected to the environments where plants are grown [2,3,4] and were found to survive up to 40 (*Salmonella*), 30 (*L. monocytogenes*), and 154 (*Escherichia coli* O157:H7) days in soil, respectively [3,5,6]. Moreover, these organisms have shown resilience under stressful plant cultivation environments with respect to water, temperature, and UV radiation [3,7,8]. At field conditions and in post-harvest and processing stages these pathogens encounter various stresses, which include but are not limited to low pH, high salt, reduced water activity, and varying temperature conditions. 

Studies reported that these stresses significantly affected the behavior and survival pattern of foodborne pathogens. *S*. *enterica* present in low water activity (a_w_) environments are considerably more tolerant to heat treatment than in high water activity environments [9]. *L. monocytogenes* and *E. coli* showed better protection against acid conditions after adaptation at low pH conditions [10,11]. Most of the microbial intervention treatment studies are focused on the treatment of bacterial cells, which were grown under normal laboratory conditions. However, it is of critical importance to understand how these stressed cells behave after a physical or chemical intervention treatment. In addition, it was reported that these treatments and stress conditions result in sub-lethally injured cells. Sub-lethally injured cells may lead to false negative results as they respond to stress and treatment by entering a physiological state which requires resuscitation [12]. The presence of sub-lethally injured cells in foods poses major public health concerns and is an important part of the assessment of the microbial response to food safety. As per our knowledge, limited data is available on the effect of commonly used inorganic and organic sanitizers such as chlorine, peracetic acid (PAA), and lactic acid on the stressed cells and the sub-lethal injury associated with it. Thus, the main objective of this study was to determine the effect of different environmental stresses on *Salmonella*, *E. coli* O157:H7, and *L. monocytogenes* response to select chemical treatments. 

## 2. Materials and Methods

### 2.1. Selection of Bacterial Strains and Inoculum Preparation

Three types of bacterial strains used in this study were *S. enterica* Typhimurium (ATCC 14028, isolated from animal tissue), *E. coli* O157:H7 (ATCC 35150, human feces isolate), and *L. monocytogenes* (ATCC 19115, Serotype 4b). All the strains were stored at −70 °C in tryptic soy broth (TSB; Hardy diagnostic, CA, USA) containing 25% glycerol (*wt*/*wt*). Prior to each replicated experiment, frozen cultures were activated by streaking onto Tryptic Soy Agar (TSA; Difco, Becton Dickinson, Sparks, MD, USA) plates and incubated at 37 °C for 18 to 24 h. Single isolated colonies from these plates were inoculated into 5 mL of TSB, followed by incubation at 37 °C with shaking for 12–14 h. These cultures were further subjected to different stresses as described below. 

### 2.2. Stress Conditions

Individual inoculums of each pathogen type were added to 100 mL of TSB at a 1:1000 dilution in 500 mL flasks. Bacterial cultures were grown to early stationary phase without shaking at 37 °C. When the pre-growth condition was either 14 or 23 °C, then the cells were grown at those respective temperatures. Growth curves were generated for each strain at each condition to determine the optical density for each growth phase at 600 nm using a UV/V is spectrophotometer (Biospectrometer, Eppendorf™, Hamburg, Germany). The cells were then subjected to six different pre-growth conditions that were found relevant to food processing and/or fresh produce environment by following Harrand et al. (2019) with modifications: (i) Acid stress: The pH of growth media was adjusted to 5 to 5.5 using Lactic acid (Fisher™, Fair Lawn, NJ, USA); (ii) Salt stress: High salt environment was generated with additional 4% NaCl (*w*/*v*); (iii) Desiccation stress: water activity was adjusted using glycerol (Fisher™, Fair Lawn, NJ, USA) at 15.6% (*v*/*v*) and 13% (*v*/*v*) to achieve an a_w_ of 0.95 ± 0.01; and (iv). Temperature stress: The cells were grown at three different temperatures (14, 23, and 37 °C). All the cells in different stress conditions were grown until the cell density reached to 10^6−7^ log CFU/mL and confirmed by the optical density measurement. 

### 2.3. Sanitizer Preparation

Three different sanitizers comprising both inorganic (sodium hypochlorite) and organic (peracetic acid and lactic acid) were studied. Two different concentrations of sodium hypochlorite (RICCA, Arlington, TX, USA) at 100 and 200 ppm were prepared using sterile deionized (DI) water. Free chlorine concentration was determined using DPD free chlorine method using DR 900 spectrophotometer (HACH™, Loveland, CO, USA). Similarly, PAA (Sanidate™ 5.0, BioSafe Systems, East Hartford, CT, USA) was prepared at 40 and 80 ppm. The concentration was determined using PAA test strips (M quant^®^, Millipore™, Darmstadt, Germany). Finally, 0.5% lactic acid solutions (*v*/*v*) were prepared using sterile DI water. These sanitizers were used immediately for conducting treatment experiments as detailed below. 

### 2.4. Treatment

The treatment procedure involves mixing 1 mL of individual inoculums separately with 9 mL of each chemical/sanitizer solution in a 15 mL centrifuge (Falcon™) tube. These samples were thoroughly mixed by four inversions and incubated for 45 s (for chlorine and PAA) and 60 s (for lactic acid). After the treatment time, the sanitizer solution (chlorine and PAA only) was immediately neutralized by adding 100 µL of 50% Na_2_S_2_O_3_ (*w*/*v*). Control samples were only subjected to treatment with phosphate-buffer saline. After the treatment, 100 μL of the appropriate dilutions were plated in duplicates on both selective (XLD for *Salmonella*, Oxford for *Listeria*, and Sorbitol MacConkey agar for *E. coli* O157:H7) and non-selective (TSA) media. Plates were incubated at 37 °C for 24 h (*S*. *enterica* and *E. coli* O157:H7) or 48 h (*Listeria monocytogenes*). The survived cells on both types of media were enumerated and expressed as log reductions (CFU/mL).

### 2.5. Sub-Lethal Injury Determination

The sub-lethal injury of pathogens was tested after each treatment. To determine sub-lethal injury rates, cells were plated onto both nonselective (TSA) and selective agars (XLD for *Salmonella*, Oxford for *Listeria*, and MacConkey agar with sorbitol for *E. coli* O157:H7). The sub-lethal injury was calculated by following method described in Estilo et al. [13]. The difference in the enumerated populations between non-selective (inured and uninjured cells) and selective (uninjured cells) media was used to calculate sub-lethally injured cells using the following equation [13,14]:

$$\%\;\mathrm{Sub}\;\mathrm{lethal}\;\mathrm{injury}=\frac{\left(\mathrm{Counts}\;\mathrm{on}\;\mathrm{Nonselective}\;\mathrm{agar}-\mathrm{Counts}\;\mathrm{on}\;\mathrm{Selective}\;\mathrm{agar}\right)\;}{\mathrm{Counts}\;\mathrm{on}\;\mathrm{Nonselective}\;\mathrm{agar}}\times 100$$

### 2.6. Statistical Analyses

All the experiments under all the conditions were repeated three times using independently grown bacterial cultures. At each sampling time within an experiment, duplicate samples were collected at the tested conditions. Data were analyzed by the analysis of variance (ANOVA) procedure using SPSS™ (Version 25, IBM^®^, Armonk, NY, USA). Tukey’s least significant difference test was used to determine the mean differences. All the tests were performed with a 0.05 level of significance.

## 3. Results and Discussion

### 3.1. Stressed Cells Response to Chlorine Treatment

**Figure 1:**
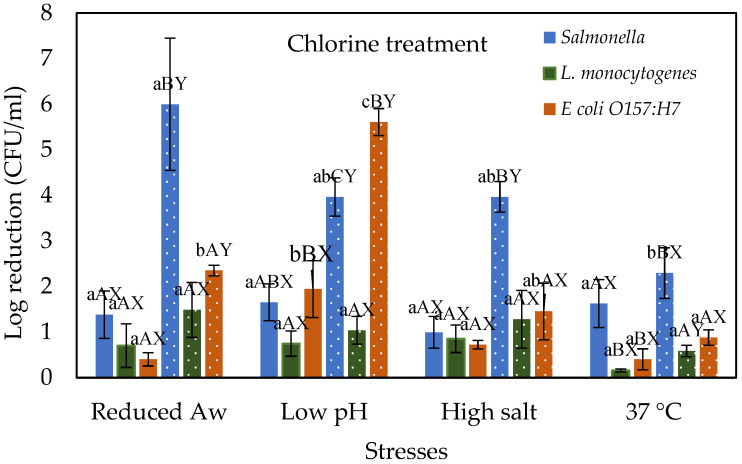
Log reductions of stressed cells of *Salmonella*, *L. monocytogenes*, *and E. coli* O157:H7 when exposed to 100 (solid) and 200 (dotted) ppm chlorine for 45 s. Different lower-case letters a, b, and c represent significant differences (*p* ≤ 0.05) in reductions between different stress conditions within one treatment and organism. Different upper-case letters A, B, C represent significant difference (*p* ≤ 0.05) in reductions between three organisms within one treatment and stress conditions. Letters X, Y, Z represent a significant difference (*p* ≤ 0.05) in reductions between treatment concentrations within same organism and stress conditions.

Figure 1 shows the effect of chlorine treatment on the log reductions of *Salmonella*, *E. coli* O157:H7, and *L. monocytogenes* cells that were prior subjected to various physiological stresses and compared with control cells grown at 37 °C. As expected, increasing the chlorine concentration from 100 to 200 ppm increased the log reductions of all tested pathogens. For example, when desiccated stressed (i.e., reduced water activity) cells of *Salmonella*, *E. coli* O157:H7, and *L. monocytogenes* subjected to chlorine treatment at 100 ppm showed a reduction of 1.38, 0.4, and 0.7 log CFU/mL, respectively. However, these reductions are not significantly different (*p* > 0.05) between the pathogens and when compared with the control cells grown at 37 °C. Further increasing the chlorine concentration to 200 ppm increased the log reductions to 6, 2.35, and 1.49 CFU/mL for *Salmonella*, *E. coli* O157:H7, and *L. monocytogenes*, respectively. *Salmonella* reduction is significantly (*p* ≤ 0.05) higher than the other pathogens. Moreover, reductions for both *Salmonella* and *E. coli* O157:H7 are significantly higher than the control while *L. monocytogenes* reductions are not different from the control. This shows better adaptability of *L. monocytogenes* under desiccation stress and subsequently less reduction to chlorine treatment at 200 ppm when compared with other test organisms. Similar results are reported by Himathongkham and Riemann [15], when *L. monocytogenes*, *Salmonella*, and *E. coli* were dried in chicken manure, the *Salmonella* and *E. coli* both decreased significantly faster than the *L. monocytogenes*. In addition, Harrand et al. [16] reported that when *Salmonella*, *L. monocytogenes*, and *E. coli* were subjected to desiccation stress *L. monocytogenes* showed better overall growth than *Salmonella* and *E. coli* under the same conditions. *Salmonella* and *E. coli* reductions are higher than *L. monocytogenes* after chlorine treatment. *Salmonella* and *E. coli* are Gram-negative, whereas *L. monocytogenes* are Gram-positive, and the Gram-positive thick peptidoglycan layer could influence susceptibility to chlorine stress. Chlorine is thought to cause bacterial cell death by impeding the functions of the inner membrane [17]. Moreover, it was also reported that inactivation by exposure to singlet oxygen is affected by the presence of the peptidoglycan layer [18].

When acid stressed (i.e., low pH) cells of *Salmonella*, *E. coli* O157:H7, and *L. monocytogenes* were treated with 100 ppm chlorine showed a reduction of 1.65, 1.94, and 0.75 log CFU/mL, respectively. The reduction of *E. coli* O157:H7 is significantly different from control cells grown under normal conditions as well as *L. monocytogenes* under acid stress. Increasing chlorine concentration to 200 ppm increased the log reductions to 3.96, 1.04, and 5.61 log CFU/mL for *Salmonella*, *L. monocytogenes*, and *E. coli* O157:H7, respectively. These reductions were significantly different between the pathogens. Moreover, *E. coli* O157:H7 reduction was significantly different from the control cells grown at 37 °C. These results indicate that Gram-negative (*Salmonella*, and *E. coli* O157:H7) are susceptible to low pH as they showed higher reductions than Gram-positive (*L. monocytogenes*). It is known that gram-negative bacteria are commonly more susceptible at low pH than Gram-positive bacteria [19]. For example, Harrand et al. [16] reported that when *L. monocytogenes* and *E. coli* were subjected to acid stress, *L. monocytogenes* showed better growth than *E. coli* under the same conditions. Similar results were observed when *E. coli* showed more sensitivity to chlorine treatment (100 ppm) than *L. monocytogenes* on lettuce leaves [20]. However, lower reductions of *L. monocytogenes* may be attributed to some inherent property of the organism itself. For example, *L. monocytogenes* cells exhibit different properties such as forming multi-chromosome filaments with buds, cell elongation, and cell wall roughness in response to chlorine stress [21,22].

When salt-stressed cells of *Salmonella*, *E. coli* O157:H7, and *L. monocytogenes* were treated with 100 ppm chlorine showed a reduction of 1, 0.72, and 0.85 log CFU/mL, respectively. No significant difference was observed between these reductions and control cells grown at 37 °C. Similarly, no significant difference among the tested pathogens under high salt stress was observed at the tested chlorine concentration. Further increasing the chlorine concentration to 200 ppm increased the log reductions to 3.97, 1.45, and 1.28 CFU/mL for *Salmonella*, *E. coli* O157:H7, and *L. monocytogenes*, respectively. *Salmonella* reduction was significantly higher (*p* ≤ 0.05) than the *E. coli* O157:H7 and *L. monocytogenes*. However, these reductions are not significantly different from the controls. The results indicated that no significant difference in reductions was observed between the tested pathogens (*p* > 0.05) and the controls after chlorine treatment. This suggests that salt stress is not likely to be a significant factor in the chlorine treatment of *Salmonella*, *E. coli* O157:H7, and *Listeria monocytogenes*.

### 3.2. Stressed Cells Response to Peracetic Acid Treatment

**Figure 2:**
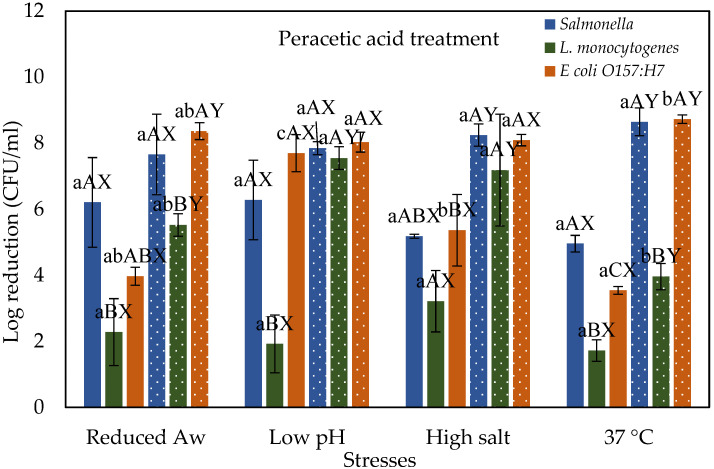
Log reductions of stressed cells of *Salmonella*, *L. monocytogenes*, *and E. coli* O157:H7 when exposed to 40 (solid) and 80 (dotted) ppm peracetic acid for 45 s. Different lower-case letters a, b, and c represent significant differences (*p* ≤ 0.05) in reductions between different stress conditions within one treatment and organism. Different upper-case letters A, B, C represent significant difference (*p* ≤ 0.05) in reductions between three organisms within one treatment and stress conditions. Letters X, Y, Z represent a significant difference (*p* ≤ 0.05) in reductions between treatment concentrations within same organism and stress conditions.

Figure 2 shows the response of different foodborne pathogens to peracetic acid (PAA) treatment when pre-subjected to various physiological stresses and compared with control cells grown at 37 °C. In general, treatment with PAA was more effective with higher log reductions than chlorine. The greater effectiveness of PAA compared to chlorine can be attributed to the strong oxidizing action of PAA that disrupts lipid and protein structures in the bacterial cell membrane causing it to lose its rigidity [23]. Also, PAA can attach to the thiol group of proteins, disrupting the functionalities of enzymes required for the synthesis of new proteins [24]. When desiccated stressed (i.e., reduced water activity) cells of *Salmonella*, *E. coli* O157:H7, and *L. monocytogenes* subjected to PAA treatment at 40 ppm showed a reduction of 6.21, 3.97, and 2.28 log CFU/mL, respectively. Further increasing the concentration to 80 ppm increased the log reductions to 7.66, 8.36, and 5.52 for *Salmonella*, *E. coli* O157:H7, and *L. monocytogenes*, respectively. Among the tested pathogens, *L. monocytogenes* reductions are significantly different from *Salmonella*. Similar results were observed by Harrand et al. [25] where desiccated *L. monocytogenes* treated by 60 ppm PAA showed less reduction compared to *Salmonella*, and *E. coli* O157:H7 after 40 ppm treatment under the same conditions. However, these reductions are not different from the control cells grown at 37 °C.

When acid stressed (i.e., low pH) cells of *Salmonella*, *E. coli* O157:H7, and *L. monocytogenes* were treated with 40 ppm PAA showed a reduction of 6.28, 7.7, and 1.92 log CFU/mL, respectively. The reduction of *E. coli* O157:H7 is significantly different from control cells grown under normal conditions. Increasing PAA concentration to 80 ppm increased the log reductions to 7.85, 7.55, and 8.03 log CFU/mL for *Salmonella*, *L. monocytogenes*, and *E. coli* O157:H7, respectively. The reduction of *E. coli* O157:H7 and *L. monocytogenes* is significantly higher than in controls grown under normal conditions. However, no significant difference among the tested pathogens under acidic conditions was observed at the tested PAA concentration. This suggests that bacterial strain under acid stress conditions is not likely to be a significant factor for reduction in a PAA treatment. However, the reduction by PAA treatment of acid stressed cells is also strain-specific, which is not explored in this study. Harrand et al. [25] reported that acid-stressed strains of these pathogens (*Salmonella*, *L. monocytogenes*, and *E. coli* O157:H7) behaved differently after PAA treatment (60 ppm for *Listeria monocytogenes,* 40 ppm for *Salmonella* and *E. coli* O157:H7). When salt-stressed cells of *Salmonella*, *E. coli* O157:H7 and *L. monocytogenes* were subjected to 40 ppm PAA showed a reduction of 5.18, 5.36, and 3.21 log CFU/mL, respectively. The reduction of *E. coli* O157:H7 is significantly different from control and other pathogens. Increasing PAA concentration to 80 ppm increased the log reductions to 8.24, 7.18, and 8.09 log CFU/mL for *Salmonella*, *L. monocytogenes*, and *E. coli* O157:H7, respectively. The reduction of *E. coli* O157:H7 and *L. monocytogenes* is significantly higher than control cells grown under normal conditions. However, no significant difference among the tested pathogens under acidic conditions was observed at the tested PAA concentration.

### 3.3. Stressed Cells Response to Lactic Acid Treatment

**Figure 3:**
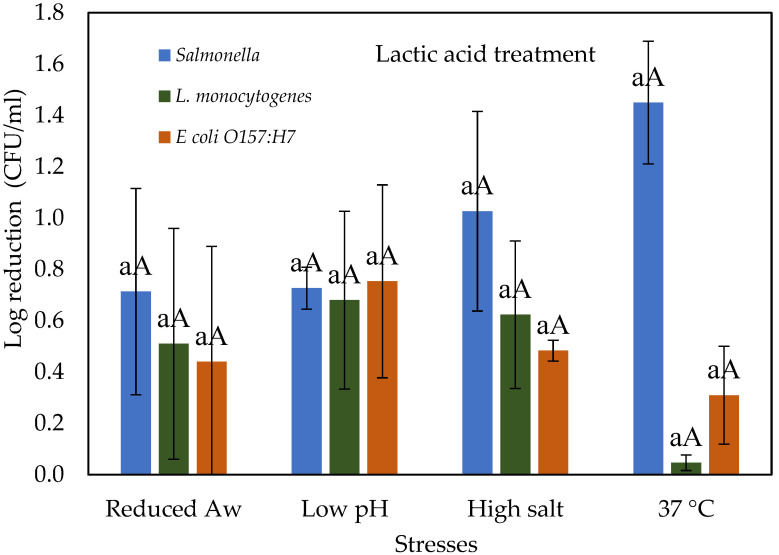
Log reduction of stressed cells of *Salmonella*, *L. monocytogenes* and *E. coli* O157:H7 when exposed to 0.5% lactic acid for 60 s. Different lower-case letter a represent significant differences (*p* < 0.05) in reductions between different stress conditions within one treatment and organism. Different upper-case letter A represent significant difference (*p* < 0.05) in reductions between three organisms within one treatment and stress conditions.

Figure 3 shows the effect of lactic acid treatment on the log reductions of *Salmonella*, *E. coli* O157:H7, and *L. monocytogenes* cells that were prior subjected to various physiological stresses and compared with control cells grown at 37 °C. The antimicrobial action of organic acids such as lactic acid is due to pH reduction in the environment, thus disrupting membrane permeability [26]. Organic acids are lipophilic and penetrate plasma membranes and thus reduce the pH of the cell interior, causing cell inactivation [27]. When desiccated, salt and acid-stressed cells of *Salmonella*, *E. coli* O157:H7, and *L. monocytogenes* subjected to lactic acid at 0.5% (*v*/*v*) showed a similar pattern of reduction of between 0.44 and 0.75 log CFU/mL. However, salt-stressed cells of *Salmonella* showed a reduction of 1.03 log CFU/mL. Similar results were observed by Sagong et al. [28] after treatment of baby spinach with 0.5% lactic acid where *Salmonella*, *E. coli* O157:H7, and *L. monocytogenes* showed a reduction of 0.91, 0.67, and 0.61 log CFU/gm. Moreover, *Salmonella* exhibited higher susceptibility, compared with that of *Listeria* and *E. coli* O157:H7. The antimicrobial susceptibility of an organism also depends on factors such as the type of microorganism and its cell membrane structure and composition [29,30]. The results indicated that no significant difference in reductions was observed among those tested food-borne pathogens (*p* > 0.05) and control cells after 0.5% lactic acid treatment. This suggests that at the tested concentration; the type of pathogen and stress conditions is not likely to be a significant factor for log reduction in an organic acid treatment.

### 3.4. Effect of Temperature Stresses on Pathogen Response to Different Chemical Treatments

**Figure 4:**
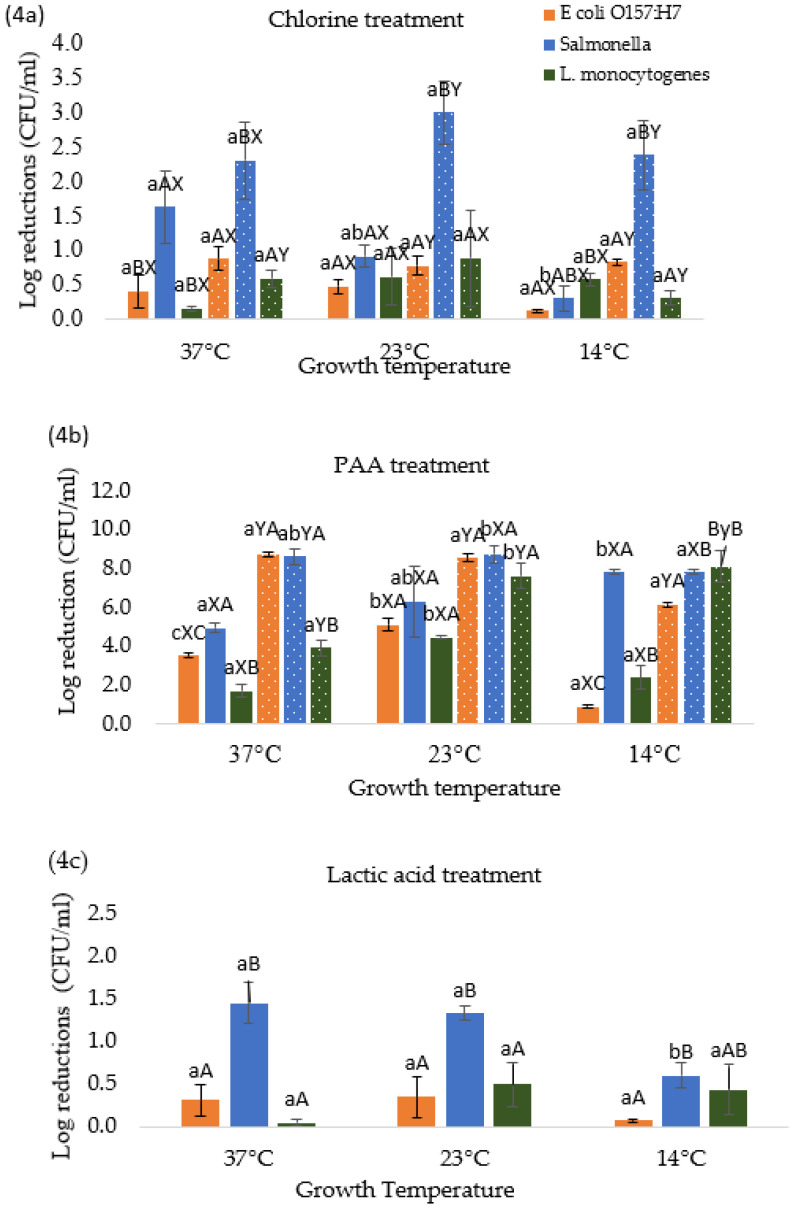
(**a**–**c**). Effect of growth temperature on the log reduction *E. coli* O157:H7, *Salmonella*, and *Listeria monocytogenes* when exposed to: (**4a**) 100 (solid) and 200 (dotted) ppm chlorine for 45 s; (**4b**) 40 (solid) and 80 (dotted) ppm peracetic acid for 45 s; and (**4c**) 0.5% Lactic acid for 60 s. Different lower-case letters a, b, and c represent significant differences (*p* < 0.05) in reductions between different stress conditions within one treatment and organism. Different upper-case letters A, B, C represent significant difference (*p* < 0.05) in reductions between three organisms within one treatment and stress conditions. Letters X, Y, Z represent a significant difference (*p* < 0.05) in reductions between treatment concentrations within same organism and stress conditions.

Figure 4a–c shows the effect of different growth temperatures on foodborne pathogens response to chlorine (Figure 4a), PAA (Figure 4b), and lactic acid (Figure 4c) treatments. When *Salmonella*, *E. coli* O157:H7, and *L. monocytogenes* were grown at room temperature 23 °C and subsequently subjected to chlorine (100 or 200 ppm) showed a reduction of 0.92, 0.48, and 0.61 log CFU/mL (at 100 ppm) and 3.00, 0.78, and 0.89 log CFU/mL (at 200 ppm), respectively (Figure 4a). It should be noted that *Salmonella* showed significantly higher reductions when compared with *E. coli* O157:H7 and *L. monocytogenes* at 23 °C for 200 ppm chlorine concentration. However, these reductions are not significantly different from the reductions observed for control cells which were grown at 37 °C (Figure 4a). Whereas decreasing the growth temperature to 14 °C reduced the reductions to 0.31, 0.12, and 0.58 log CFU/mL (for chlorine at 100 ppm) and 2.38, 0.83, and 0.31 log CFU/mL (for chlorine at 200 ppm) for *Salmonella*, *E. coli* O157:H7 and *L. monocytogenes*, respectively. These results show pathogen specific variations in response to chlorine treatment with respect to growth temperatures. Moreover, the reductions at 14 °C were lower as compared to cells grown at 23 and 37 °C indicating that the efficacy of the chlorine treatment against cells grown at 23 and 37 °C was higher than at 14 °C. This can be attributed to a higher metabolic activity of bacteria at 23 and 37 °C than at 14 °C, so they were more easily affected by the action of the sanitizers [31]. Similar results were observed by Al-Nabulsi et al. [32] in a study where *E. coli* showed fewer reductions at 10 °C than 25 °C when rocket salad leaves were treated with chlorine.

Figure 4b shows the effect of PAA treatment when the cells were pre-grown at 14, 23, and 37 °C. At 23 °C, the reductions were found to be significantly higher (*p* ≤ 0.05) for *E. coli* O157:H7 (at 40 ppm) and *L. monocytogenes* (at 40 and 80 ppm) when compared with the control cells at 37 °C. Similar results were observed by Harrand et al. [25] when *L. monocytogenes* stressed at 21 °C showed higher reductions than control at 37 °C. However, no significant difference among the tested pathogens under room temperature growth conditions was observed at tested PAA concentrations. Further decreasing the growth temperature to 14 °C resulted in a reduction of 7.83/7.83 (*Salmonella*), 0.93/6.17 (*E. coli* O157:H7), and 2.39/8.14 (*L. monocytogenes*) log CFU/mL, at 40/80 ppm of PAA treatment, respectively. Depending upon the PAA concentration, reductions were significantly different between the cells grown at 14 and 37 °C (control). Moreover, a significant difference was observed among the tested pathogens at 14 °C temperature and the tested PAA concentrations.

Figure 4c shows the response of different foodborne pathogens to lactic acid treatment when subjected to temperature stresses and compared with control (37 °C). When *Salmonella*, *E. coli* O157:H7, and *L. monocytogenes* were stressed at room temperature (23 °C) and subsequently subjected to lactic acid treatment showed a reduction of 1.33, 0.35, and 0.49 log CFU/mL, respectively. However, at 14 °C, *Salmonella*, *E. coli* O157:H7, and *L. monocytogenes* showed lower reductions of 0.60, 0.07, and 0.44 log CFU/mL, respectively. *Salmonella* showed significantly higher reduction and appeared to be more sensitive to lactic acid than *E. coli* O157:H7 and *L. monocytogenes*. This may be attributed to the fact that *Salmonella* cells size decreases significantly than *E. coli* O157:H7 and *L. monocytogenes* after treatment with 0.5% lactic acid [33]. Similar results were also observed by Akbas and Ölmez [34], Ban et al. [35] after treatment with 2% lactic acid on PVC, lettuce, and steel surfaces. Moreover, at 14 °C reductions are lower than 37 and 23 °C this may also be because metabolic activities of bacteria are high at 23 and 37 °C than at 14 °C.

### 3.5. Sub-Lethal Injury Determination

The sub-lethal injury was determined as the fraction of the population exposed to treatments that can form colonies on the non-selective agar, but not on the selective agar. Table 1 shows the sub-lethal injury in stressed *Salmonella*, *E. coli*, and *L. monocytogenes* after different chemical treatments. Sub-lethal injury typically affects cell wall or membrane permeability (structural injury) but may also cause extensive damage to various functional cell components [36]. Due to this damage, injured cells showed different nutritional and physiological needs which causes an inability to form colonies on selective media. This inability is the result of membrane damage [13,37]. In terms of treatment, the highest injured cells were observed on PAA treatments. As PAA has a high oxidizing capacity [23]. However, as for stress conditions, desiccation for *Salmonella* and low temperature (14 °C) for *E. coli* and *L. monocytogenes* showed the highest injured cells. Finn et al. [38] reported that *Salmonella* showed better adaptability to low moisture conditions. *Salmonella* responds to desiccation stress by various mechanisms, including biofilm formation, accumulation of solutes, filamentation of cells, and rRNA degradation [38,39,40]. On the other hand, *E. coli* and *L. monocytogenes* responses to cold stress include the synthesis of cold shock proteins which were involved in a variety of essential functions such as transcription, translation, mRNA degradation, protein synthesis, and recombination [8,41]. 

## 4. Conclusions

This study evaluated the effect of different environmental stresses on *Salmonella*, *E. coli* O157:H7, and *L. monocytogenes* response when exposed to commonly used inorganic and organic sanitizers. Our results indicated that different stress conditions have a varied effect on the organisms when subjected to these treatments. However, the effect of the tested environmental stresses varies from pathogen to pathogen. In general, stressed cells of *Salmonella* showed highest sensitivity while *Listeria* showed the least sensitivity to tested treatments. Low temperature (14 °C) growth conditions showed significantly different behavior of the tested pathogens when compared to commonly followed laboratory conditions of growth at 37 °C. This study highlights that the pre-growth conditions of pathogens were critical to assessing the efficiency of chemical treatments in the food industry. Future research should be focused on determining the effect of stress conditions on the growth kinetics of pathogens on different foods and may provide new insights into the behavior of these stressed pathogens. Furthermore, what role the environmental stresses or sub lethally injuries play in the biofilm environment need be to explored.

## Figures and Tables

**Table 1:** Calculation of percent sub-lethally injured cells of tested pathogens after subjecting to different treatments.

Type of Organism	Type of Stress Conditions	Growth Conditions				Treatment and % Injury				
		pH	aw	Temp (°C)	Time (h)	Chlorine Treatment		Lactic Acid	Peracetic Acid Treatment	
						100 ppm	200 ppm	0.5%	40 ppm	80 ppm
*S.* enterica	Control	7.2	0.99	37	18	2.80 ± 1.1	4.62 ± 2.90	−4.62 ± 6.15	14.78 ± 13.88	0.00 ± 0.00
	Acid	5	0.99	37	18	6.16 ± 4.32	6.15 ± 4.99	1.23 ± 0.18	16.00 ± 21.33	0.00 ± 0.00
	Desiccation	7.2	0.96	37	18	6.95 ± 5.38	2.00 ± 1.81	−0.70 ± 6.96	5.95 ± 5.95	33.33 ± 57.74
	High salt	7.3	0.99	37	18	2.47 ± 1.64	8.45 ± 0.68	19.06 ± 7.07	1.97 ± 10.11	0.00 ± 0.00
	Ambient Temp	7.2	0.99	23	18	2.08 ± 1.32	3.77 ± 1.57	5.43 ± 6.67	17.61 ± 27.70	0.00 ± 0.00
	Low Temperature	7.2	0.99	14	70	0.20 ± 2.27	3.28 ± 3.32	−2.07 ± 3.37	0.00 ± 0.00	0.00 ± 0.00
*L. monocytogenes*	Control	7.2	0.99	37	18	2.70 ± 0.85	4.62 ± 1.28	1.61 ± 0.57	8.13 ± 6.47	64.80 ± 32.25
	Acid	5.5	0.99	37	18	3.79 ± 3.16	0.81 ± 1.48	−0.03 ± 3.15	58.51 ± 27.03	0.00 ± 0.00
	Desiccation	7.2	0.95	37	18	3.67 ± 0.32	2.15 ± 4.42	1.08 ± 0.96	15.62 ± 3.90	64.13 ± 6.54
	High salt	7.3	0.99	37	18	1.24 ± 0.86	4.07 ± 0.65	−0.32 ± 1.86	9.24 ± 10.33	20.11 ± 17.41
	Ambient Temp	7.2	0.99	23	18	−0.55 ± 2.32	1.29 ± 0.36	0.04 ± 2.63	7.57 ± 14.45	66.67 ± 57.74
	Low Temperature	7.2	0.99	14	70	1.42 ± 0.73	5.55 ± 1.06	3.10 ± 2.42	25.41 ± 16.78	66.67 ± 57.74
*E. coli* O157:H7	Control	7.2	0.99	37	18	2.49 ± 1.82	3.95 ± 2.25	1.85 ± 1.17	18.30 ± 21.26	0.00 ± 0.00
	Acid	5	0.99	37	18	0.12 ± 1.72	7.93 ± 3.90	0.30 ± 0.30	0.00 ± 0.00	0.00 ± 0.00
	Desiccation	7.2	0.96	37	18	4.22 ± 3.06	5.17 ± 2.34	−1.50 ± 1.85	12.92 ± 14.57	0.00 ± 0.00
	High salt	7.3	0.99	37	18	−1.03 ± 0.55	20.43 ± 4.02	−1.16 ± 1.24	86.38 ± 23.59	0.00 ± 0.00
	Ambient Temp	7.2	0.99	23	18	0.60 ± 1.02	0.85 ± 1.56	0.98 ± 1.09	1.65 ± 3.12	0.00 ± 0.00
	Low Temperature	7.2	0.99	14	70	0.28 ± 0.10	0.24 ± 0.60	0.50 ± 0.62	3.30 ± 2.81	32.52 ± 15.17

## Data Availability

The data presented in this study are available in the article.
